# CHI3L1 induces autophagy through the JNK pathway in lung cancer cells

**DOI:** 10.1038/s41598-023-36844-4

**Published:** 2023-06-20

**Authors:** Da Eun Hong, Ji Eun Yu, Seung Sik Yoo, In Jun Yeo, Dong Ju Son, Jaesuk Yun, Sang-Bae Han, Jin Tae Hong

**Affiliations:** College of Pharmacy and Medical Research Center, Chungbuk National University, 194-31, Osongsaengmyeong 1-ro, Osong-eup, Cheongju-si, Chungbuk 28160 Republic of Korea

**Keywords:** Cancer, Cell biology

## Abstract

CHI3L1 is closely related to the molecular mechanisms of cancer cell migration, growth, and death. According to recent research, autophagy regulates tumor growth during various stages of cancer development. This study examined the association between CHI3L1 and autophagy in human lung cancer cells. In CHI3L1-overexpressing lung cancer cells, the expression of LC3, an autophagosome marker, and the accumulation of LC3 puncta increased. In contrast, CHI3L1 depletion in lung cancer cells decreased the formation of autophagosomes. Additionally, CHI3L1 overexpression promoted the formation of autophagosomes in various cancer cell lines: it also increased the co-localization of LC3 and the lysosome marker protein LAMP-1, indicating an increase in the production of autolysosomes. In mechanism study, CHI3L1 promotes autophagy via activation of JNK signaling. JNK may be crucial for CHI3L1-induced autophagy since pretreatment with the JNK inhibitor reduced the autophagic effect. Consistent with the in vitro model, the expression of autophagy-related proteins was downregulated in the tumor tissues of CHI3L1-knockout mice. Furthermore, the expression of autophagy-related proteins and CHI3L1 increased in lung cancer tissues compared with normal lung tissues. These findings show that CHI3L1-induced autophagy is triggered by JNK signals and that CHI3L1-induced autophagy could be a novel therapeutic approach to lung cancer.

## Introduction

Chitinase-3-like protein-1 (CHI3L1) is a glycoprotein expressed in mononuclear cells, macrophages, neutrophils, cultured cartilage cells, and synovial cells. It is a member of the glycoside hydrolase 18 family and is crucial for cell division, migration, and inflammation^1^. In addition, CHI3L1 is highly expressed in leukemia, lymphoma, sepsis, coronary artery and lung disease, which indicates a poor prognosis^2–4^. According to previously published studies, CHI3L1 expression increases in the serum and lungs of patients with idiopathic pulmonary fibrosis^5^, high serum CHI3L1 level in small-cell lung cancer patients is associated with early death^6^, and serum CHI3L1 levels in non-small-cell lung cancer and melanoma patients are used as independent pre-biomarkers^7^. Our previous study reported that lung metastasis was suppressed in CHI3L1-knockout mice or CHI3L1 inhibitor use in mice^8^. In addition, several studies have shown that CHI3L1 knockdown inhibits lung cancer cell proliferation and promotes cancer cell death^9–11^.

Cell death is largely classified into apoptotic cell death, necrosis, and autophagic cell death. Apoptosis or programmed cell death removes unwanted cells during early stage cancer development. It plays an important role in the clinical treatment of human cancer, which is regulated by many cell death-related genes and signaling pathways. Apoptosis is regulated by intracellular or extracellular signals and is characterized by morphological changes in target cells including nuclear fragmentation and condensation, mitochondrial membrane permeation (MOMP), membrane blisters, cell contraction, and cell apoptosis^12–15^.On the other hand, autophagy is a “self-eating” mechanism by which cells are degraded and cell molecules and organelles are regenerate^16^. It is a proteolytic process that occurs in cells to maintain homeostasis against various cell stresses and is caused by changes in the external environment, such as protein disorders, incorrect folding stress, nutritional deficiency, growth factor reduction, and ER stress. Autophagy acts as an intermediate agent in cancer, aging, and inflammatory reactions^17–20^. The role of autophagy in cancer is very complicated and not yet fully understood. Autophagy can play a positive or negative role in promoting cancer cell death. It can act as a tumor-suppression mechanism to defend against tissue damage, cell damage, and inflammation and has been shown to cause autophagy-dependent cell death by excessively degrading the cytoplasm^15,21,22^. Autophagy can also act as a tumor-promotion mechanism by supplying energy to cancer cells, during which cells lose the ability to control cell damage and become malignant as tumors form and metastasize^23^. Numerous studies have suggested an association between autophagy and the development of cancer^24^.

According to many studies on the biological mechanisms of autophagy, autophagy is complex in both human health and diseases. Many proteins are known to control autophagy-related tumorigenesis. Increased CHI3L1 level is correlated with poor prognosis and decreased survival rate in cancer patients. In addition, CHI3L1 depletion has been reported to inhibit tumorigenesis. Based on these results, this study investigated the effect of high expression of CHI3L1 on autophagy in lung cancer cells and its basic molecular mechanisms.

## Methods

### Cell culture

A549 and H460 NSCLC cells were obtained from the American Type Culture Collection (Manassas, VA, USA). Cells were cultured in RPMI 1640 medium supplemented with 10% heat-inactivated fetal bovine serum (FBS), 100 μg/mL penicillin, and 100 μg/mL streptomycin. Cell cultures were maintained in an incubator with a humidified atmosphere of 5% CO_2_ at 37 °C. Cell culture was performed as described previously.

### Transfection

For transfection, A549 and H460 cells were transiently transfected with pcDNA 3.1-CHI3L1 plasmid or CHI3L1 siRNA, using Lipofectamine 3000 (for plasmid DNA) (Invitrogen, Carlsbad, CA) and RNAiMAX (for siRNA) reagent, according the manufacturer’s protocol. Transfection was performed using Opti-MEM medium with 1 µg/ml plasmid DNA or 20 µM siRNA. Another mixture with transfection reagent was prepared. Both of the mixture solution were mixed together and incubated for 20 min. The complex solution was added to cells and the cells were incubated for 1–2 days with a humidified atmosphere of 5% CO_2_ at 37 °C.

### Cell lysates preparation and Western blotting

Cell lysates and Western blotting was performed as described previously^25^. Transfected cells were washed with cold-PBS and harvested with lysis buffer containing 50 mM Tris–HCl (pH 7.5), 0.25 mM NaCl, 0.1% Triton X-100, and 2 mM EDTA with protease inhibitor and phosphatase inhibitor. The obtained cell lysates were then placed on ice for 30 min and were centrifuged at 12,000× *g* for 30 min at 4 °C. In total, 10 μg of proteins was subjected to SDS-PAGE for separation and then transferred on PVDF membranes. After blocking the membranes with 5% bovine serum albumin (BSA) in PBS for 1 h, the membranes were incubated with specific primary antibodies and subsequently with HRP-conjugated secondary antibodies. The desired proteins were detected using an enhanced chemiluminescence substrate (#WBKLS0500, Millipore, Billerica, MA) and visualized using the FUSION Solo S chemiluminescence detection system (Vilber Lourmat, Collégien, France).

### Immunocytochemistry

Immunocytochemistry was performed as described previously^26^. A549 and H460 cells were cultured in coverslips and transfected. The cells were washed with PBS and fixed using 4% paraformaldehyde for 15 min at room temperature. The cells were then permeabilized with cold-methanol for 5 min and blocked using 4% BSA in PBS with 0.1% Triton X-100 (PBS-T) for 1 h. The primary antibodies with appropriate dilutions were added, and the cells were incubated overnight at 4 °C. After washing 3 times with PBS-T, coverslips were incubated with Alexa Fluor 488 (#A32723, #A32731, Invitrogen, Carlsbad, CA) or Texas Red (#T-862, #T-2767, Invitrogen)-conjugated secondary antibodies for 1 h at room temperature. The cells were then incubated with 1 μg/ml DAPI (#D9542, Sigma-Aldrich, St. Louis, MO) for 5 min at room temperature and subsequently mounted using the Fluoromount-G Mounting Medium (#0100-01, Southern Biotech, Birmingham, AL). The cells were visualized using the ZEISS Axio Observer fluorescence microscope system (Carl Zeiss, Oberkochen, Germany). Digital images were analyzed using ZEN 2.1 software (Carl Zeiss). For quantitative analysis, 50 cells were counted in 10 random fields and performed three independent experiments.

### Autophagy flux assay

According to the manufacturer's protocol, the autophagic flux was assessed using the CYTO-ID^®^ Autophagy detection kit (Enzo Life Sciences Inc., Farmingdale, NY). Transfected cells were washed with 1× assay buffer, and then stained with CYTO-ID Green dye for 1 h at 37 °C in the dark. The cells were then incubated with 1 μg/ml DAPI (#D9542, Sigma-Aldrich, St. Louis, MO) for 5 min at room temperature and subsequently mounted using the Fluoromount-G Mounting Medium (#0100-01, Southern Biotech, Birmingham, AL). The cells were visualized the ZEISS Axio Observer fluorescence microscope system (Carl Zeiss). Digital images were analyzed using ZEN 2.1 software (Carl Zeiss).

### Human sample

Human tissue samples from lung cancer tissue and normal controls were obtained from Chungbuk National University Hospital Biobank, Keimyung University Dongsan Hospital Biobank and the Biobank of Ajou University Hospital, members of Korea Biobank Network. All studies using human samples were conducted in accordance with the Declaration of Helsinki and were approved by the Ethics Committee of Chungbuk National University Medical Center (CBNU-201910-BR-941-01). The experiments were undertaken with the understanding, informed consent, and written consent of each subject.

### Animal sample

Mice were maintained under controlled conditions of temperature and light. They were provided standard mice feed and water ad libitum. Animal expreriments and procedures was carried out in compliance with the ARRIVE guidelines and all protocols involving mice in this study were reviewed and approved by the Chungbuk National University Institutional Animal Care and Use Committee (IACUC) and complied with the Korean National Institute of Health Guide for the Care and Use of Laboratory Animals (CBNUA-792-15-01). Animal experiments were conducted in accordance with relevant guidelines and regulations.

### *Xenograft* animal model

*Xenograft* animal model samples were used as described previously^27^. A549 human lung cancer cells were injected subcutaneously (s.c.) (1 × 10^7^ cells/100 μL in PBS/animal) into upper dorsal region of the mice. Implantation tumors visually detected in injected region of the mice after 5–7 days. The tumor growth of the mice were monitored twice per week during 4 weeks. At the end of the experiment, the animals were sacrificed using a CO2 gas chamber and the tumors were separated from the surrounding muscles and dermis, and excised.

### Immunohistochemisty (IHC)

IHC was performed as described previously^8^. For IHC, the CHI3L1-WT and –KO mice tumor tissue sections were blocked with 3% normal goat or horse serum diluted in PBS, for 30 min; the sections were then incubated with antibodies for CHI3L1, p-JNK, LC3, p62 and LAMP-1 at the appropriate dilution in blocking serum, for overnight at 4 °C. The slides were washed in PBS, followed by the avidin–biotin–peroxidase complex (ABC) (#PK-6101, Vector Laboratories, Burlingame, CA, USA). The slides were washed, and the peroxidase reaction was developed with diaminobenzidine and peroxide (#SK-4100, Vector Laboratories), mounted in Aqua-Mount, and evaluated under a light microscope (Olympus).

### Immunofluorescence

The CHI3L1-WT and -KO mice tumor tissue sections were blocked with 3% normal goat and horse serum diluted in PBS, for 30 min; the sections were then incubated with antibodies for LC3 and LAMP-1, at the appropriate dilution in blocking serum, for overnight at 4 °C. The slides were washed in PBS, and incubated with Alexa Fluor 488 (#A32723, #A32731, Invitrogen, Carlsbad, CA) or Texas Red (#T-862, #T-2767, Invitrogen)-conjugated secondary antibodies for 1 h at room temperature. The slides were then incubated with 1 μg/ml DAPI (#D9542, Sigma-Aldrich, St. Louis, MO) for 5 min at room temperature and subsequently mounted (#0100-01, Southern Biotech, Birmingham, AL). The slides were visualized using the ZEISS Axio Observer fluorescence microscope system (Carl Zeiss, Oberkochen, Germany). Digital images were analyzed using ZEN 2.1 software (Carl Zeiss).

### Cell invasion assay

For the trans-well assay, the A549 and H460 cells were seeded on upper chamber inserts (8 μm pore trans-well; Corning Inc., New York, NY, USA). The lung cancer cells were plated at 2.0 × 10^4^ cells per well and incubated at 37 °C, 5% CO_2_, in a humidified incubator. The cells were then fixed with 4% formaldehyde for 5 min and permeated with 100% methanol for 15 min before being stained with 0.1% crystal violet for 20 min. In the upper chamber, non-migrated cells were removed with a cotton swab. Using an Olympus light microscope, migrated cell pictures were observed and analyzed using ImageJ software (NIH).

### TUNEL assay

According to the manufacturer’s instructions, the DeadEnd™ Fluorometric TUNEL System (Promega, Madison, Wisconsin, WI, USA) was used for the TUNEL assay to detect apoptotic cells. Lung cancer cells (2 × 10^4^ cells/well) were cultured on 8-chamber slides. The cells were washed with PBS and fixed by 4% paraformaldehyde in PBS for 20 min at room temperature. They were then permeabilized by 0.1% Triton X-100 in PBS for 5 min at room temperature. The slides were incubated with mounting media for fluorescence-containing DAPI (Vector Laboratories, Inc., Burlingame, CA, USA) for 15 min, at room temperature and in the dark. After this, the cells were visualized using the ZEISS Axio Observer fluorescence microscope system (Carl Zeiss, Oberkochen, Germany). Digital images were analyzed using ZEN 2.1 software (Carl Zeiss).

### Statistical analysis

Statistical analysis was performed as described previously^28^. Statistical analyses were performed using the GraphPad Prism 5 software (GraphPad software, Inc., San Diego, CA, USA). All error bars reported are the standard deviation (SD) unless otherwise indicated. Pairwise comparisons were performed using Student’s t-test. Multiple comparisons were using one-way analysis of variance followed by Tukey’s tests. Differences between groups were considered significant at *P* values of < 0.05.

## Results

### CHI3L1 induced autophagosome formation in lung cancer cells

**Figure 1:**
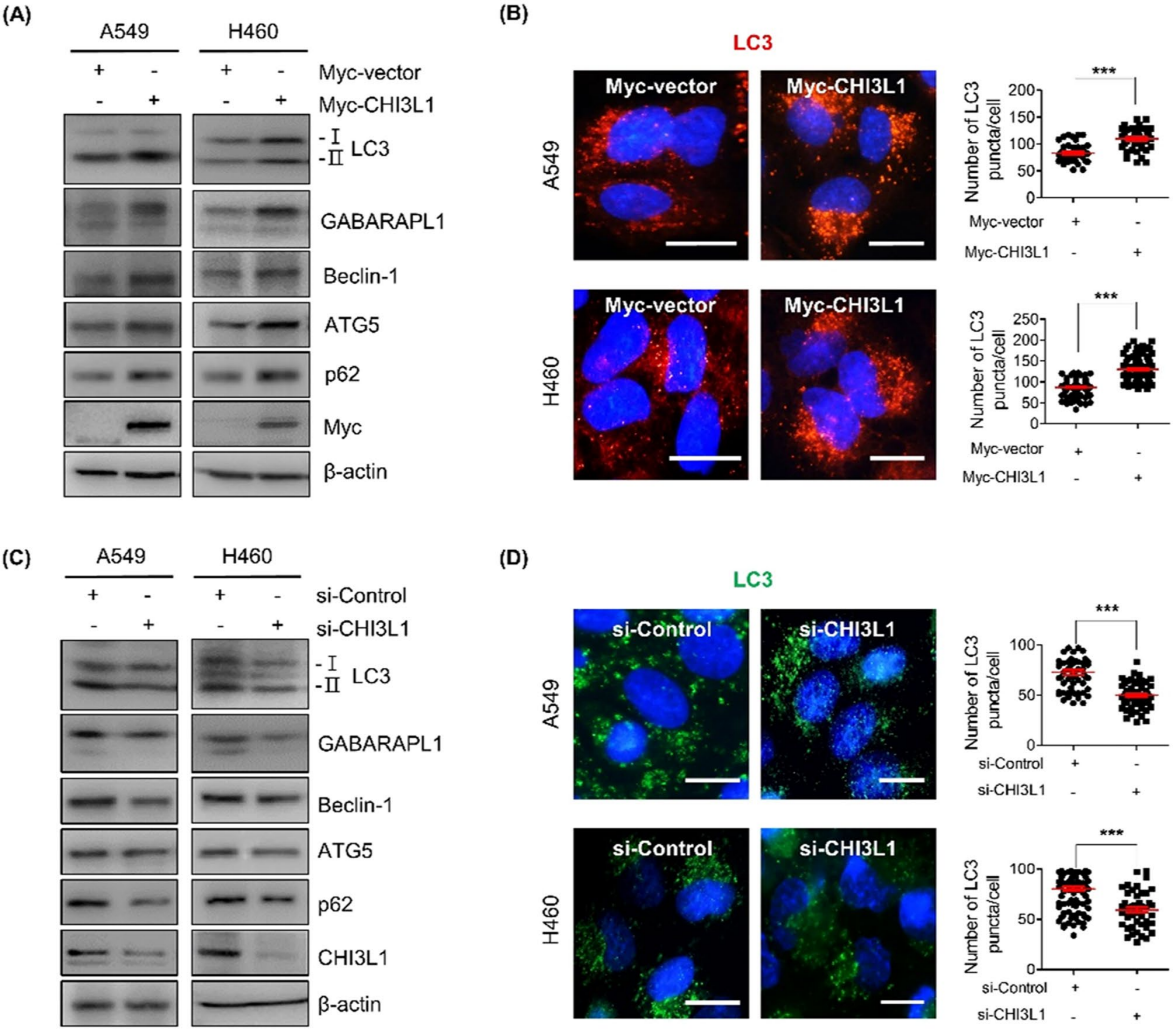
CHI3L1 enhances formation of autophagosomes in human lung cancer cells. (**A**) A549 and H460 cells were transfected with either Myc-vector or Myc-CHI3L1 for 24 h. The expression of autophagosome-related proteins such as LC3, GABARAPL1, Beclin-1, ATG5 and p62 was evaluated by Western blotting. (**B**) Transfected cells were stained with LC3 antibody. Using fluorescent microscopy, LC3 puncta formation was detected. The number of LC3 puncta per cell was calculated. The data was the average of three independent experiment and error bars were mean ± SD. ***, *p* < 0.001. Scale bar, 10 μm. (**C**) A549 and H460 cells were transfected with either siRNA Control or CHI3L1 siRNA for 48 h. The expression of autophagosome-related proteins such as LC3, GABARAPL1, Beclin-1, ATG5 and p62 was evaluated by Western blotting. (**D**) Transfected cells were stained with LC3 antibody. Using fluorescent microscopy, LC3 puncta formation was detected. The number of LC3 puncta per cell was calculated. The data was the average of three independent experiment and error bars were mean ± SD. ***, *p* < 0.001. Scale bar, 10 μm.

Several studies have reported that autophagy has a role in both the development and suppression of cancer and promotes tumorigenesis by preferentially recognizing particular targets like damaged organs, protein aggregates, and intracellular pathogens^29^. Our previous study has demonstrated that CHI3L1 promotes cell growth^30^. Although several proteins have reportedly been involved in autophagy processes, studies on the role and mechanisms of CHI3L1 are unknown. This study attempted to investigate the effect of CHI3L1 on autophagy in lung cancer. The expression of the autophagosome marker protein LC3 was confirmed to determine whether CHI3L1 induces autophagy in lung cancer cell lines including A549 and H460 cells. Western blot analysis revealed that CHI3L1 overexpression increased the conversion from LC3-I to LC3-II, followed by the expression of GABARAPL1, ATG5, p62, and Beclin-1 (Fig. 1A). We also confirmed the mRNA levels of autophagy-related genes using RT-qPCR. *LC3*, *Beclin-1*, *ATG5* and *p62* mRNA levels were increased in CHI3L1 overexpressed A549 and H460 cells (Supplementary Fig. S1A). In addition, we investigated whether CHI3L1 overexpression induced LC3 puncta formation by fluorescent staining of A549 and H460 lung cancer cells with LC3 antibodies. We found that CHI3L1 overexpression significantly increased LC3 puncta accumulation in lung cancer cells (Fig. 1B). To confirm whether autophagosomes were increased in other cell lines, LC3 expression was confirmed in liver cancer and colon cancer cell lines. CHI3L1 overexpression increased the expression of LC3-II and LC3 puncta formation in Hep3B and SW480 cancer cell lines (Supplementary Fig. S1B, S1C). Conversely, CHIL31 depletion decreased the conversion from LC3-I to LC3-II in lung cancer cell lines as well as the expression of GABARAPL1, ATG5, p62, and Beclin-1 (Fig. 1C). The mRNA levels of *LC3*, *Beclin-1*, *ATG5*, and *p62* were also decreased by CHI3L1 depletion (Supplementary Fig. S1D). Furthermore, CHI3L1 depletion decreased LC3 puncta formation in lung cancer cell lines (Fig. 1D). These data indicate that CHI3L1 overexpression induced autophagy in various cancer cell lines, especially lung cancer cells.

### CHI3L1 induced autolysosome formation in lung cancer cells

**Figure 2:**
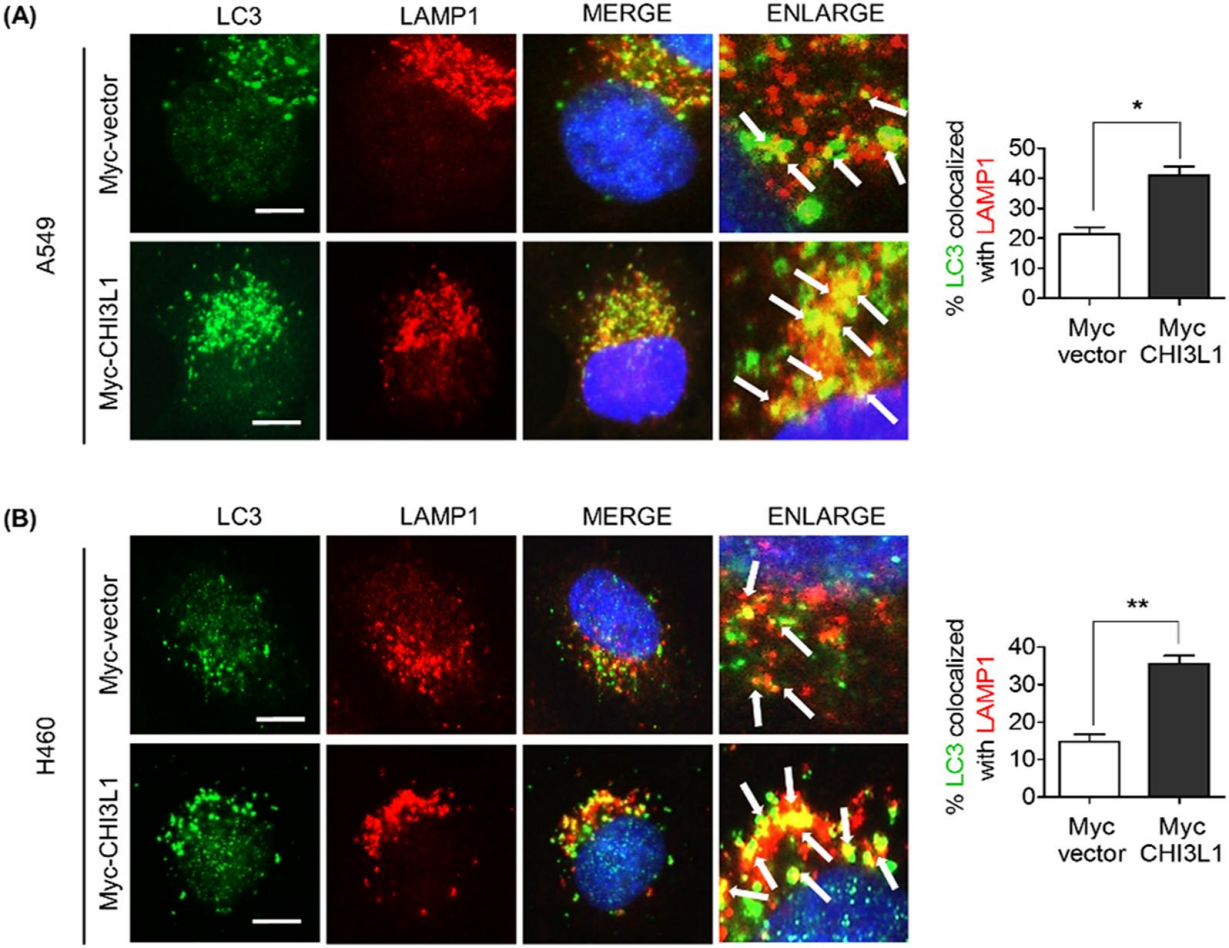
CHI3L1 enhances formation of autolysosomes in human lung cancer cells. (**A**) A549 and (**B**) H460 cells were transfected with either Myc-vector or Myc-CHI3L1 for 24 h. The cells were fixed and permeabilized. Cell were immunostained with LC3 (Green) and LAMP-1 (Red) during fusion with autophagosome and lysosome. Cell nucleus was stained with Hoechst 33,342 (blue). Autolysosome localization observed by fluorescence microscopy. The data was the average of three independent experiment and error bars were mean ± SD. *, *p* < 0.05, **, *p* < 0.01. Scale bar, 10 μm.

Autophagy describes the degradation process that occurs after autolysosome and autophagosome fusion with lysosomes. We investigated the expression of the late endosome/lysosome marker protein LAMP-1 to further examine the induction of autophagy. Western blot results showed no significant change in the LAMP-1 protein level (Supplementary Fig. S2). Additionally, LAMP-1 staining revealed no significant differences in fluorescent intensity. As shown in Fig. 2, most of the LC3 puncta were not associated with LAMP-1 in Myc-vector-overexpressing cells. However, increased LC3 puncta was co-localized with LAMP-1 in Myc-CHI3L1-overexpressing cells, indicating that CHI3L1 induces autolysosomal formation. Lysosomes are dynamic organelles, and their intracellular position changes in response to various treatments^31^. Our results can predict that CHI3L1 overexpression does not affect LAMP-1 expression and promotes lysosome recruitment with autophagosomes, resulting in increased autolysosome fusion. Thus, we provide evidence that CHI3L1 causes autophagy induction by increasing the formation of autolysosomes and autophagosomes.

### CHI3L1 induced autophagy flux in lung cancer cells

**Figure 3:**
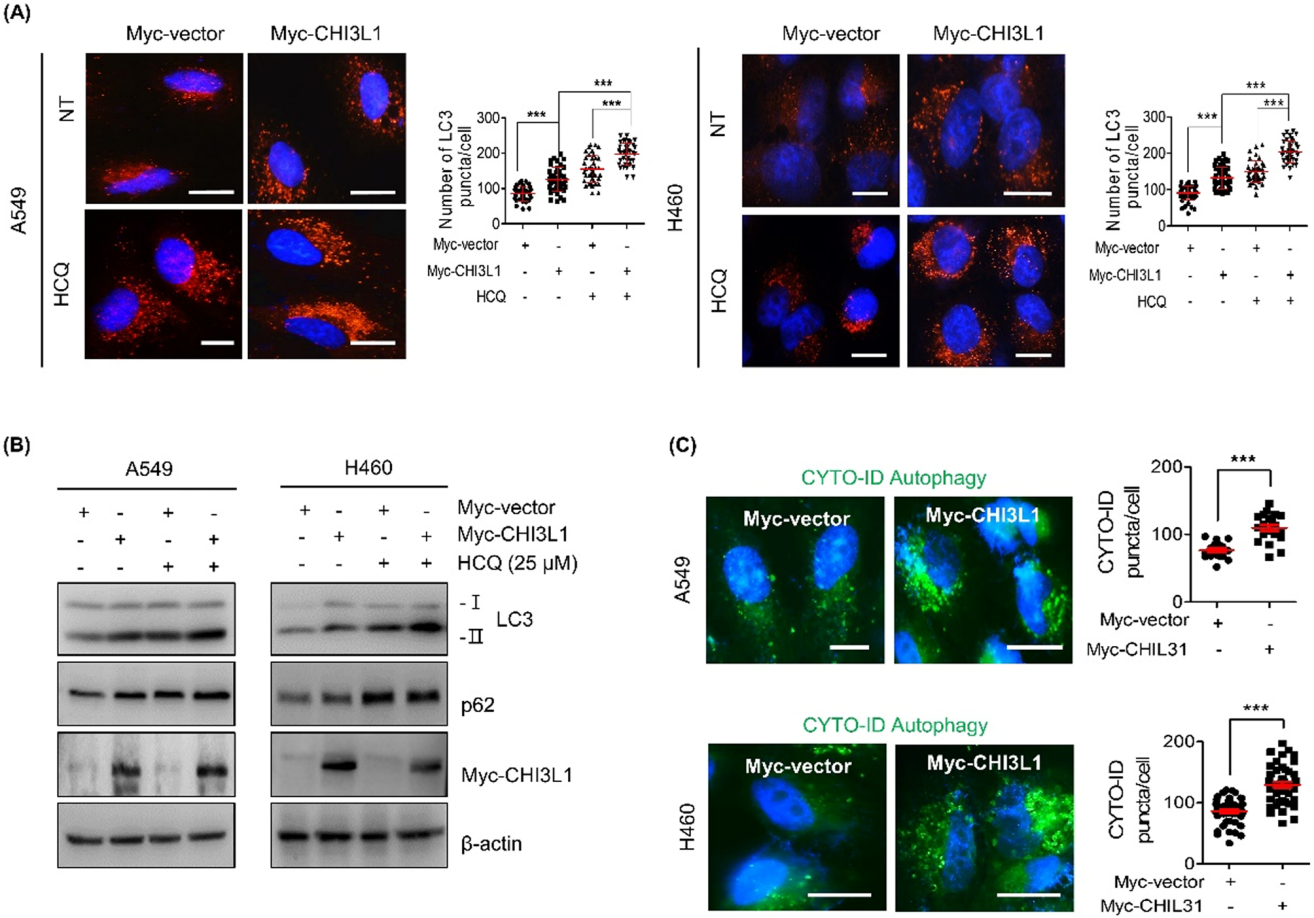
CHI3L1 induces autophagic flux in human lung cancer cells. (**A**) A549 and H460 cells were transfected with either Myc-vector or Myc-CHI3L1 for 24 h with or without HCQ (25 μM) for 6 h. The cells were fixed, permeabilized and then stained with LC3 antibody. Using fluorescent microscopy, LC3 puncta formation was detected. The number of LC3 puncta per cell was calculated. The data was the average of three independent experiment and error bars were mean ± SD. ***, *p* < 0.001. Scale bar, 10 μm. (**B**) The expression of LC3 and p62 levels were evaluated by Western blotting. (**C**) Transfected cells were stained with CYTO-ID for 1 h at 37 °C in the dark. Cell nucleus was stained with Hoechst 33,342 (blue). The data was the average of three independent experiment and error bars were mean ± SD. ***, *p* < 0.001. Scale bar, 10 μm.

The formation of autophagosomes and LC3 level in the autophagic compartment can both be increased via autophagy induction and inhibition. To clarify these two possibilities, the autophagic flux was examined. Autophagy flux represents the synthesis of autophagosomes, transfer of autophagic substrates to lysosomes, and degradation of fused autophagic substrates with lysosomes. Autophagy flux is considered a more reliable indicator of autophagy activation^32^. Hydroxychloroquine (HCQ), an autophagy inhibitor, enhances LC3-II levels by blocking the fusion of autophagosomes with lysosomes. Myc-CHI3L1 plasmid DNA was transfected in lung cancer cells with or without HCQ to determine whether CHI3L1 overexpression induces autophagy flux. After treatment with Myc-CHI3L1 or HCQ alone, LC3-II levels increased. Combination treatment with Myc-CHI3L1 and HCQ further increased LC3-II levels (Fig. 3B). In addition, Myc-CHI3L1-transfected lung cancer cells showed an extensive distribution of fluorescent LC3 puncta patterns, which were further increased with HCQ treatment (Fig. 3A). However, CHI3L1 depletion resulted in decreased LC3-II level. Compared with CHI3L1 siRNA transfection or HCQ treatment, combination treatment with CHI3L1 siRNA and HCQ decreased LC3-II expression (Supplementary Fig. 3A). LC3 puncta was decreased in only siRNA CHI3L1-transfected lung cancer cells and also decreased with combination treatment with siRNA CHI3L1 and HCQ compared with siRNA control and HCQ (Supplementary Fig. 3B). These results showed that CHI3L1 depletion inhibits autophagy. Next, we used the CYTO-ID^®^ autophagy detection kit, which is another method for measuring autophagy flux. The CYTO-ID^®^ autophagy detection kit selectively labels accumulated autophagic vacuoles by monitoring the autophagy flux staining probe. Results showed that Myc-CHI3L1 induced more autophagy fluxes than Myc-vector, indicating that the probe only stained active autophagy fluxes. Further, the number of CYTO-ID-positive cells increased in Myc-CHI3L1-overexpressing cells (Fig. 3C). Collectively, we demonstrated that CHI3L1 overexpression induces autophagy.

### CHI3L1 induced autophagy through activation of the JNK pathway

**Figure 4:**
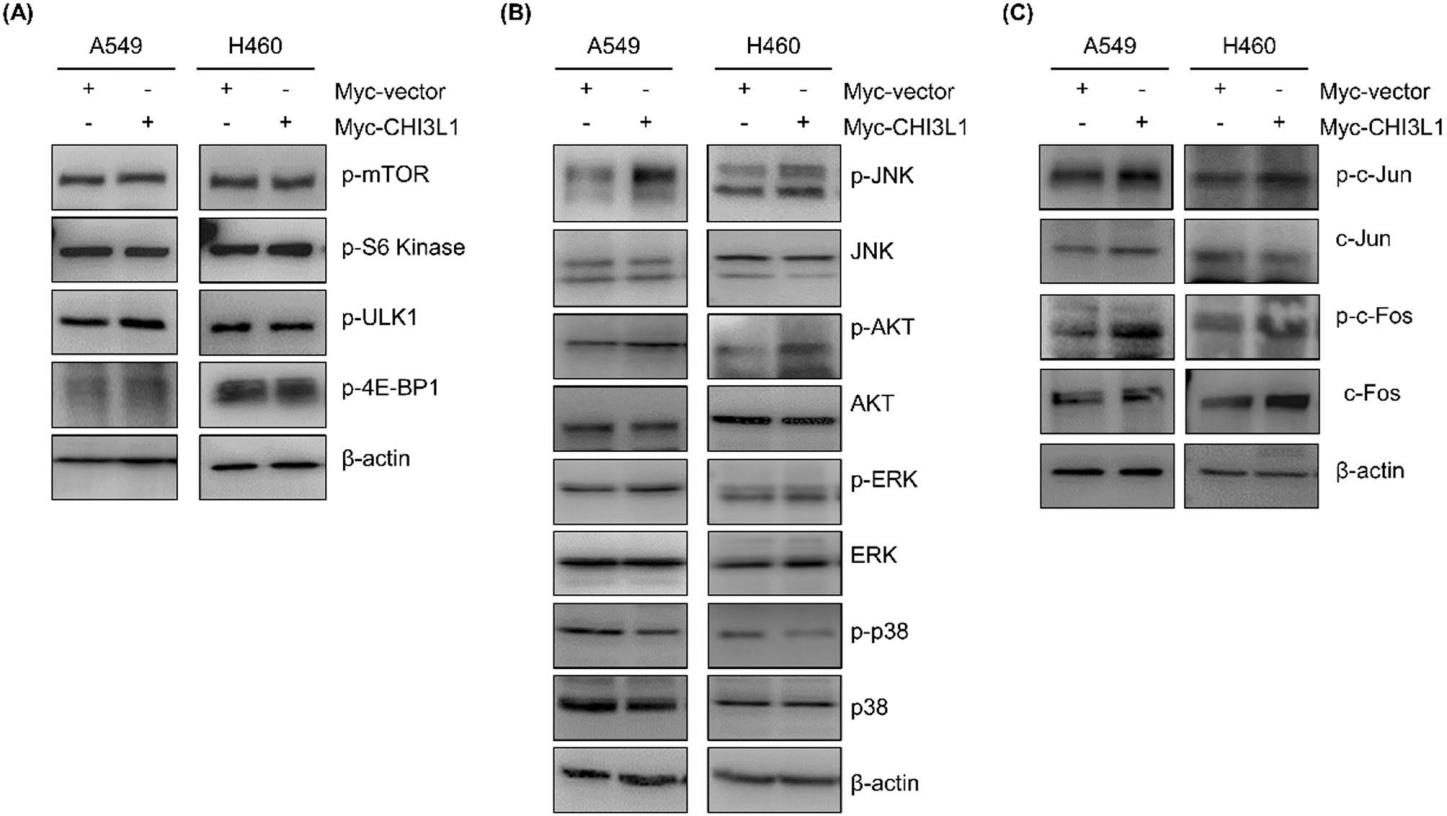
Activation of the JNK pathway involved in CHI3L1-induced autophagy. A549 and H460 cells were transfected with either Myc-vector or Myc-CHI3L1 for 24 h. (**A**) The expression of mTOR pathway related proteins such as p-mTOR, p-S6 kinase, p-ULK1 and p-4E-BP1 was evaluated by Western blotting. (**B**) The expression of MAPK pathway related proteins such as p-JNK, JNK, p-AKT, AKT, p-ERK, ERK, p-p38, and p38 was evaluated by Western blotting. (**C**) The expression of JNK downstream pathway related proteins such as p-c-Jun, c-Jun, p-c-Fos, and c-Fos was evaluated by Western blotting. Western blotting was performed two times with duplicate samples.

**Figure 5:**
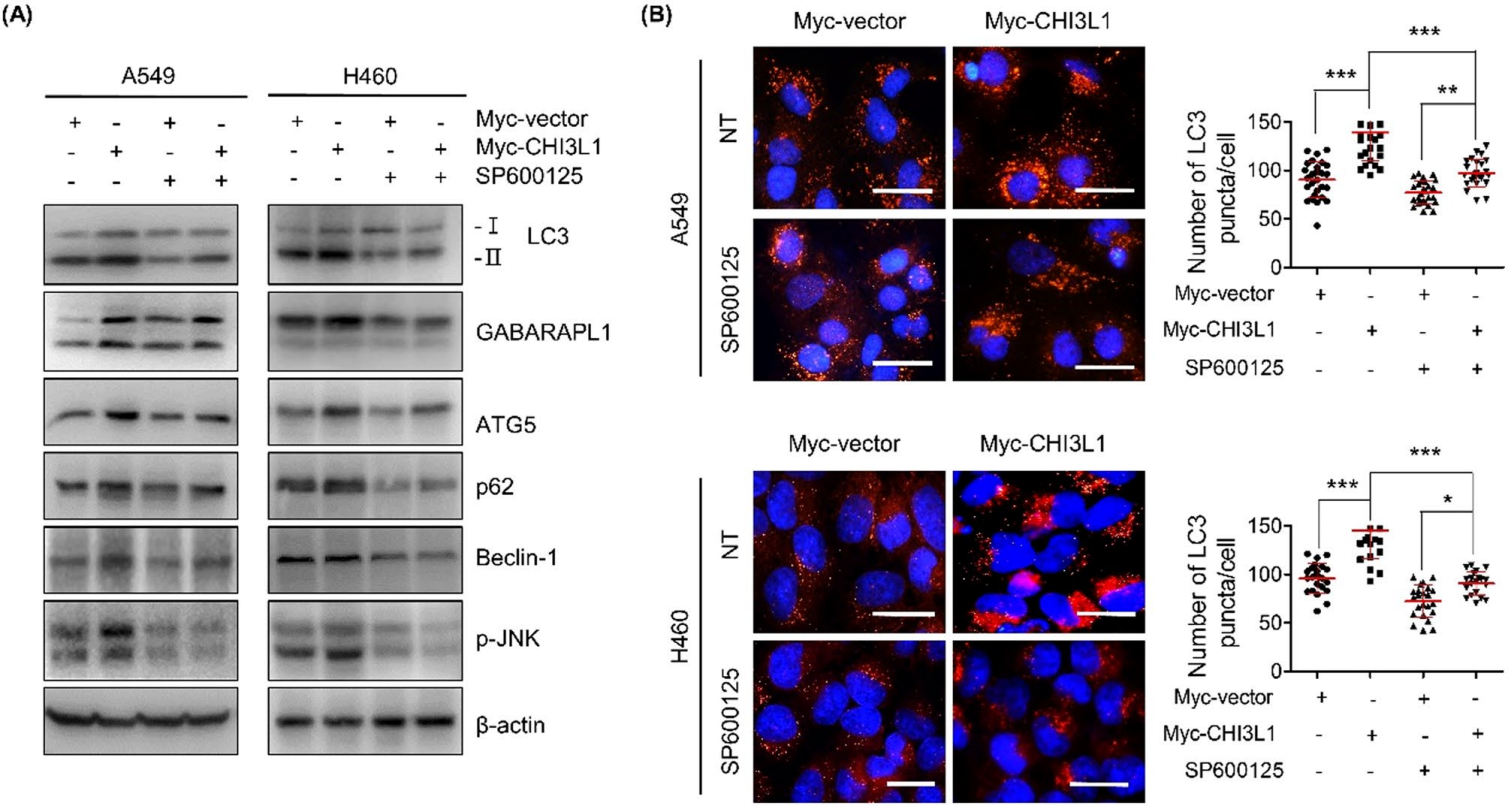
Blocking of JNK signaling inhibited CHI3L1-induced autophagy. (**A**) A549 and H460 cells were transfected with either Myc-vector or Myc-CHI3L1 for 24 h with or without JNK inhibitor, SP600125, pre-treatment (20 μM) for 2 h. The indicated protein expression levels were evaluated by Western blotting. (**B**) Transfected cells were fixed, permeabilized and then stained with LC3 antibody. Using fluorescent microscopy, LC3 puncta formation was detected. The number of LC3 puncta per cell was calculated. The data was the average of three independent experiment and error bars were mean ± SD. ***, *p* < 0.001; **, *p* < 0.01; *, *p* < 0.05. Scale bar, 20 μm.

Autophagy is mostly induced by signal suppression of the mammalian target of rapamycin (mTOR). We first confirmed whether CHI3L1 induced autophagy through the mTOR signaling pathway and investigated the expression of mTOR and the eukaryotic translation initiation factor 4E-bounding protein 1 (4E-BP1), ribosomal protein S6 kinase beta-1 (p70S6K), and Unc-51 like autophagy activating kinase (ULK), known for mTOR downstream signaling. Unexpectedly, CHI3L1 overexpression had no effect on the level of mTOR phosphorylation and its downstream targets 4E-BP1, p70S6K, and ULK (Fig. 4A). This result suggested that CHI3L1-induced autophagy mechanism was not dependent on mTOR signaling. MAPK/JNK signaling cascade is well known to be involved in the regulation of autophagy^33,34^. Therefore, we confirmed the expression of MAPK pathway-related proteins (JNK, AKT, ERK, and p38). Among them, JNK phosphorylation increased in CHI3L1-overexpressing cells (Fig. 4B). JNK is phosphorylated to activate c-Jun/c-Fos, which triggers autophagy by increasing the transcriptional activity of Beclin-1 and the transcriptional regulatory activity of the ATG gene^35,36^. We investigated whether CHI3L1 overexpression induces autophagy by activating JNK signal. We confirmed that JNK phosphorylation was elevated in CHI3L1-overepxressing lung cancer cells. Moreover, CHI3L1 overexpression increased the phosphorylation of c-Jun and c-Fos (Fig. 4C). To verify that CHI3L1 actually induces autophagy via JNK signaling activation, we investigated CHI3L1-induced autophagy on treatment with the JNK inhibitor SP600125. LC3 expression, which was increased by CHI3L1 overexpression, was decreased with SP600125 treatment (Fig. 5A). These results were again confirmed with LC3 puncta formation. CHI3L1 overexpression considerably increased the formation of LC3 puncta. However, LC3 puncta decreased on combination treatment with SP600125 and Myc-CHI3L1 (Fig. 5B).

In cancer, autophagy can have both pro-survival and pro-death effects. To investigate whether CHI3L1-induced autophagy by JNK activation affects cancer cell proliferation, we performed MTT assay. CHI3L1 overexpression increased the cell survival. However, when JNK signaling is inhibited by SP600125 treatment, CHI3L1 overexpression induced cell viability was prevented in lung cancer cells (Supplementary Fig. S4A). To determine whether JNK depeldent CHI3L1-induced autophagy resulted in the inhibition of the lung cancer cell invasion, we performed trans-well assay. CHI3L1 overexpression increased the invasion in lung cancer cells. However, the combination treatment with SP600125 and CHI3L1 overexpression reduced the cell invasion compared with CHI3L1 overexpression alone (Supplementary Fig. S4B). In addition, we confirmed the effect of JNK-mediated autophagy by overexpression of CHI3L1 resulted in cell apoptosis by performing TUNEL assay. The combination treatment of SP600125 and CHI3L1 overexpression further increased apoptotic cells compared with CHI3L1 overexpression alone (Supplementary Fig. S4B). These result suggest that CHI3L1 overexpression promote lung cancer cell proliferation and invasion through JNK signaling-induced autophagy.

### CHI3L1-induced autophagy in vivo

**Figure 6:**
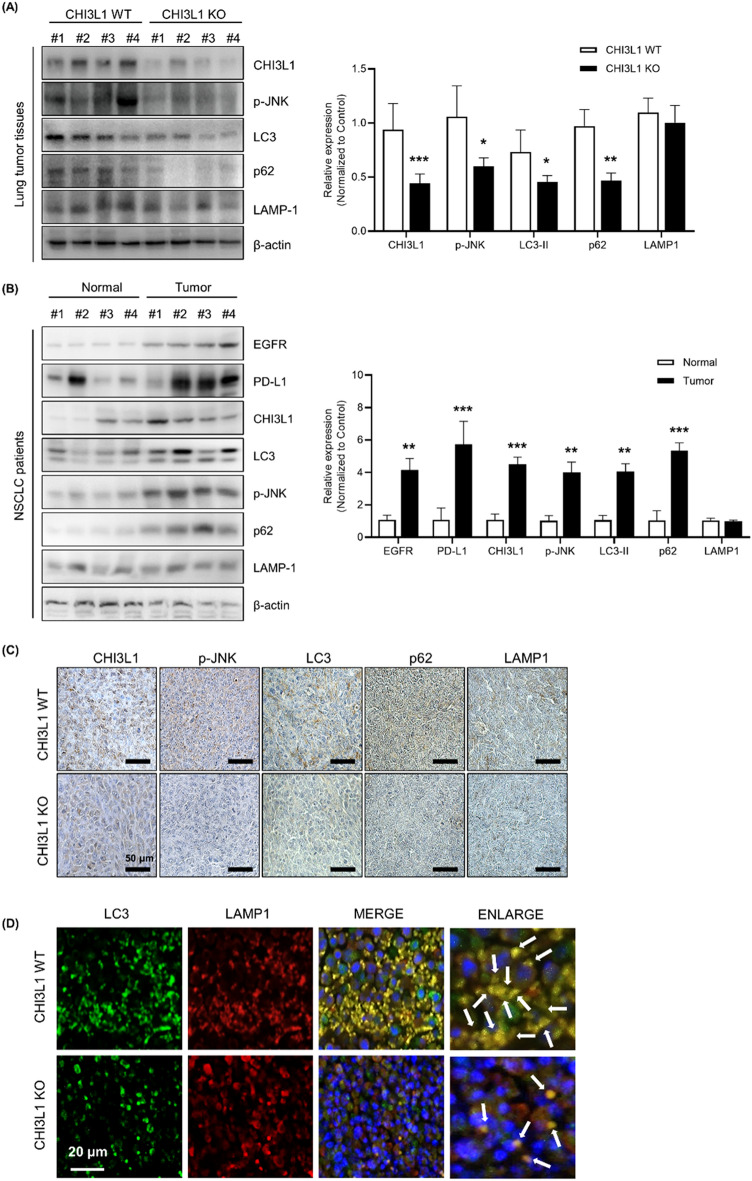
CHI3L1-induced autophagy in vivo. (**A**) A549 human lung cancer cells were subcutaneously injected in CHI3L1-WT and -KO mice, and the formation of xenografted tumors was confirmed later. After 4 weeks, they were sacrificed to obtain tumor tissues. The indicated protein expression levels were evaluated by Western blotting. The relative protein expression in the CHI3L1-KO mice tumor tissue group compared with that in the control group is shown in the graphs. ***, *p* < 0.001; **, *p* < 0.01; *, *p* < 0.05 (vs. Control). (**B**) The indicated protein expression levels from human patients tissue lysates was evaluated by Western blotting. The relative protein expression in the NSCLC human patients group compared with that in the control group is shown in the graphs. ***, *p* < 0.001; **, *p* < 0.01 (vs. Control). (**C**) Immunohistochemical analysis for CHI3L1, p-JNK, LC3, p62, and LAMP-1 in CHI3L1-WT and -KO mice tumor tissue sections. Scale bar, 50 μm. (**D**) Immunofluorescence analysis for anti-LC3 and anti-LAMP-1 in CHI3L1-WT and -KO mice tumor tissue sections. The sections were immunostained with LC3 (Green) and LAMP-1 (Red) during fusion with autophagosome and lysosome. Cell nucleus was stained with Hoechst 33,342 (blue). The data was the average of three independent experiment. Scale bar, 20 μm.

Next, we analyzed the change in the expression of CHI3L1 and autophagy-related proteins using human lung cancer tissues. As a result, the expression of autophagy-related proteins LC3 and p62 increased in lung cancer tissues compared with normal lung tissues. Furthermore, phosphorylated JNK and CHI3L1 expression also increased in tumor tissues (Fig. 6A). Our previous studies revealed that lung tumor growth was suppressed in CHI3L1-KO mice. The expression of autophagy-related proteins was confirmed in the lung tumor tissues of CHI3L1-WT and -KO mice. Autophagy-related proteins were confirmed in CHIL31-WT/KO mice with human lung cancer xenografts. Western blot results showed that the expressions of CHI3L1, p-JNK, LC3, and p62 in CHI3L1-KO lung tumor tissue decreased compared with those in CHI3L1-WT lung tumor tissues. Similar to in vitro results, there was no change in LMAP-1 expression in lung tumor tissues of CHI3L1-WT and -KO mice, suggesting that CHI3L1 did not affect lysosomal expression (Fig. 6B). Immunohistochemical analysis results showed that the expression of autophagy-related proteins and phosphorylated JNK was also decreased in CHI3L1-KO lung tumor tissues (Fig. 6C). Additionally, autolysosome formation was confirmed in CHI3L1-WT and -KO lung tumor tissues using co-staining of LC3 and LAMP-1. As a result, LC3 puncta was decreased in CHI3L1-KO mice. In addition, the number of LC3 and LAMP-1 co-localized cells also decreased in CHI3L1-KO tumor tissues (Fig. 6D). These results showed that CHI3L1-KO inhibits autophagy in in vivo models. CHI3L1 inhibition is expected to suppress lung tumorigenesis via autophagy inhibition.

## Discussion

In this study, we found that CHI3L1 induces autophagy in lung cancer cell by increasing the formation of autophagosomes and autolysosomes. We also demonstrate that the JNK pathway is related to CHI3L1-induced autophagy. In addition, we confirmed that autophagy was suppressed in lung tumor tissues of CHI3L1-KO mice as in in vivo model.

CHI3L1 is used as a biomarker for inflammatory diseases, early detection of neuro inflammatory and disease diagnosis, and prognosis for cancers^1,37^. Many studies have shown that decreased expression of CHI3L1 is effective in treating cancer cells by inhibiting the metastasis of lung cancer and inducing apoptosis^9–11^. The potential role and mechanisms of CHI3L1 in tumorigenesis and disease development are considered important. Autophagy plays a role in cancer initiation, damaged cell repair, cell survival, and cell death induction. Autophagy can promote autophagic cell death, and its inhibition can lead to cell death and a greater degree of cancer cell death^38^. However, the molecular mechanism by which CHI3L1 regulates autophagy and is involved in lung cancer is unknown. Based on these results, the effect and mechanism of CHI3L1 on autophagy in lung cancer were investigated. In this study, we found that LC3 expression and LC3 puncta formation increased in CHI3L1-overexpressing lung cancer cells. Also, CHI3L1 overexpression induced autophagy in various cancer cell lines such as human liver cancer and colon cancer cell lines. Conversely, CHI3L1 depletion inhibited the formation of autophagosomes.

Autophagy is induced by various stresses, and intracellular waste products or dysfunctional organelles are isolated by autophagosomes. Autophagosomes fuse with late endosomes or lysosomes to become autolysosomes, which then cause ubiquitinated cargo material degradation^39^. We confirmed that CHI3L1 overexpression does not affect the expression of lysosomes and induces fusion between autophagosomes and lysosomes through co-localization with the lysosomal marker LAMP-1. Our data showed that CHI3L1 induces autophagosome–lysosome fusion, but its detailed mechanism remains unclear. We hypothesized on the mechanism of CHI3L1 with autophagosome–lysosome fusion for autolysosomes formation. Kinesin-dependent transportation is essential for the accurate location of autophagosomes, predicting that CHI3L1 can affect intracellular membrane tracking through the Rab, GTPase, and tethering complex to induce autophagosomes and lysosome locational shift via dynein/dynactin-driven movement. Based on this hypothesis, CHI3L1 overexpression is expected to shift the lysosome position to autophagosomes. These results show that CHI3L1 induces the formation of autolysosomes, leading to autophagy^40^.

The most well-known induction signal in autophagy is mTOR. However, our results showed that CHI3L1 does not affect mTOR activation. Autophagy can be induced through signals such as JNK, AKT, and Ras, in mTOR-independent signal^41^. We confirm that the phosphorylation of JNK and JNK downstream factors also increased in CHI3L1-overexpressing cells. In addition, JNK inhibition with SP600125 decreased the levels of the autophagy marker protein LC3-II and formation rate of LC3 puncta in A549 and H460 cells. Furthermore, our results showed that CHI3L1 overexpression induces cell proliferation and invasion in lung cancer cells. However, under SP600125 treatment, CHI3L1 overexpression-induced cell viability was prevented in lung cancer cells. These results suggests that blocking the JNK signaling pathway in CHI3L1 overexpressed-lung cancer cells may have the inducing effect on apoptosis, while inhibiting effects of autophagy. The pro-survival effects of CHI3L1-induced autophagy may be counteracted by the pro-apoptotic effects of JNK inhibition in lung cancer cells. These results indicated that activation of the JNK pathway by CHI3L1 is essential for inducing autophagy.

Autophagy plays a dual-edged role in cancer, as it can prevent tumor necrosis and promote tumor initiation in the early stages of the disease^42^. It was also confirmed using lung tumor tissues from patients. As a result, lung tumor tissues had much higher levels of autophagy-related proteins expression than normal lung tissues. In addition, we confirmed that autophagy-related proteins and the fusion of LC3 and LAMP-1 decreased in CHI3L1-KO mice lung tumor tissues. In our previous study, the expression of cell cycle related-, apoptosis related-, and proliferation related-proteins in CHI3L1-KO mice were controlled to reduce lung metastasis^9^. In addition, many studies on autophagy that promote tumor progression are currently being studied. Studies have shown that tumor-specific PD1 and PD-L1 improve tumor progression by increasing autophagy through interaction with ATG13^43^. Malat1 proved useful in finding a more effective treatment for colon cancer as autophagy activation by miR-101 expression inhibition in cancer cell lines promotes cell proliferation and reduces apoptosis^44^. Taken all together, CHI3L1 depletion is expected to reduce the ability of autophagy to promote tumor metastasis.

Therefore, our findings suggest a novel mechanism for the induction and signaling of autophagy by CHI3L1 in lung cancer and suggest that autophagy inhibition by CHI3L1 can be a novel therapeutic application for lung cancers.

## Supplementary Information

Supplementary Information.

## Data Availability

The authors declare that all data supporting the findings in this study are available within the paper, Supplementary information and Source data. All data are available upon request to the corresponding author.
