# Supplementary material for: CHI3L1 induces autophagy through the JNK pathway in lung cancer cells
Source: Sci Rep. 2023 Jun 20;13:9964. doi: 10.1038/s41598-023-36844-4 (PMC10281972; doi:10.1038/s41598-023-36844-4)
Supplement: Supplementary file 1 — Supplementary Information. [file 41598_2023_36844_MOESM1_ESM.docx]

**Supporting information**

**CHI3L1 induces autophagy through the JNK pathway in lung cancer cells**

**Da Eun Hong^1,*^, Ji Eun Yu^1,*^, Seung Sik Yoo^1^, In Jun Yeo^1^, Dong Ju Son^1^, Jaesuk Yun^1^, Sang-Bae Han^1,#^, Jin Tae Hong^1,#^**

^1^College of Pharmacy and Medical Research Center, Chungbuk National University, 194-31, Osongsaengmyeong 1-ro, Osong-eup, Cheongju-si, Chungbuk 28160, Republic of Korea.

^*^: These authors contributed equally to this work.

**# *Corresponding authors*:** Drs. Jin Tae Hong (jinthong@chungbuk.ac.kr) and Sang Bae Han (shan@chungbuk.ac.kr), College of Pharmacy and Medical Research Center, Chungbuk National University, Osongsaengmyeong 1-ro, Osong-eup, Heungdeok-gu, Cheongiu, Chungbuk, 28160, Republic of Korea, Tel: +82-42-261-2813, Fax: +82-42-268-2732

**
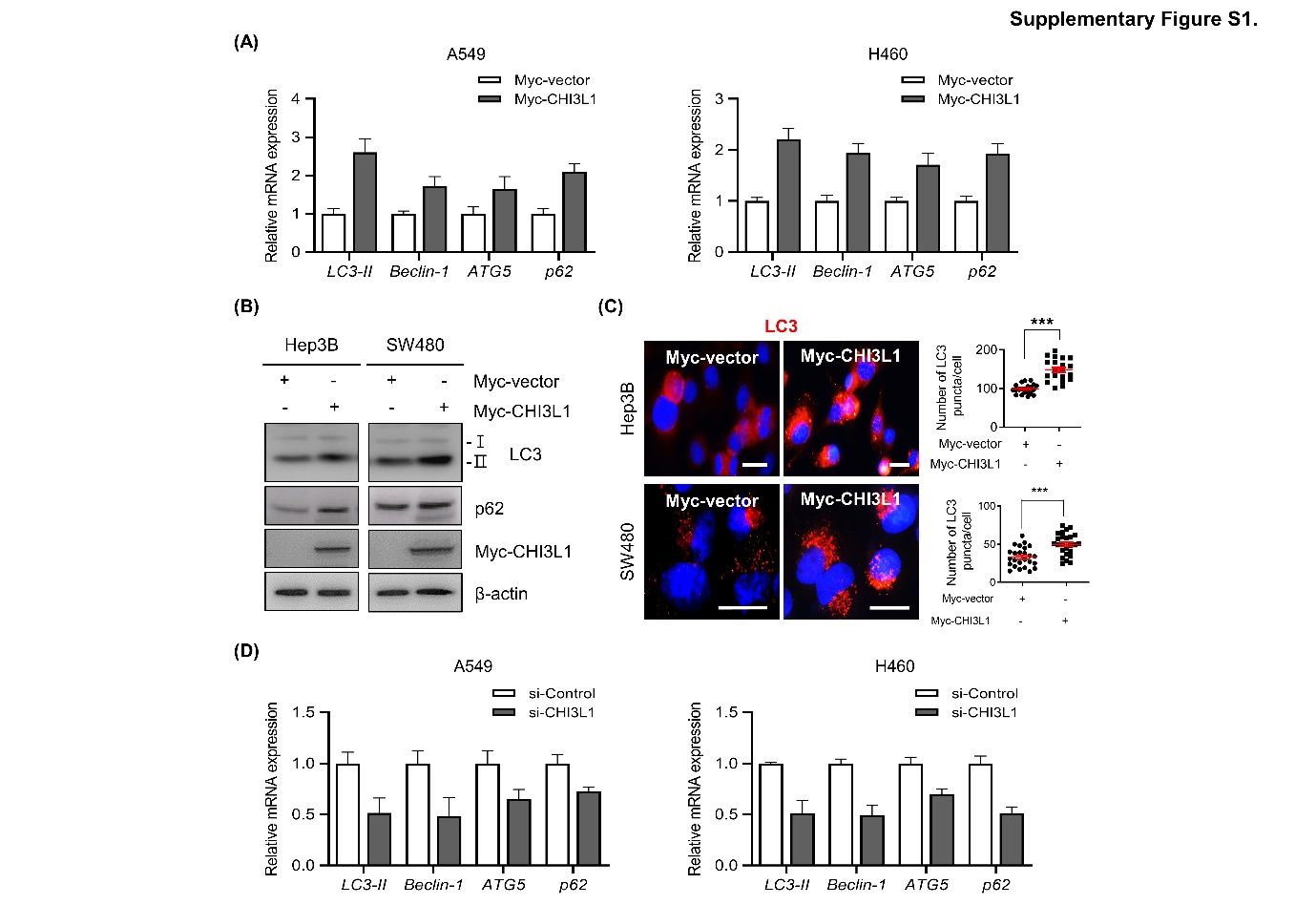
**

**Supplementary Figure S1. CHI3L1 enhances formation of autophagosome in various cancer cell lines.** (**A**) A549 and H460 cells were transfected with either Myc-vector or Myc-CHI3L1 for 24 h. The mRNA level of autophagosome-related genes such as *LC3, Beclin-1, ATG5* and *p62* was evaluated by RT-qPCR. (**B**) Liver cancer cell line, Hep3B and colon adenocarcinoma cell line, SW480 cells were transfected with either Myc-vector or Myc-CHI3L1 for 24 h. The expression of LC3 and p62 was evaluated by Western blotting. (**C**) Transfected cells were stained with LC3 antibody. Using fluorescent microscopy, LC3 puncta formation was detected. The number of LC3 puncta per cell was calculated. The data was the average of three independent experiment and error bars were mean ± SD. ***, p<0.001. Scale bar, 10 μm. (**D**) A549 and H460 cells were transfected with either siRNA Control or CHI3L1 siRNA for 48 h. The mRNA level of autophagosome-related genes such as *LC3, Beclin-1, ATG5* and *p62* was evaluated by RT-qPCR.

**
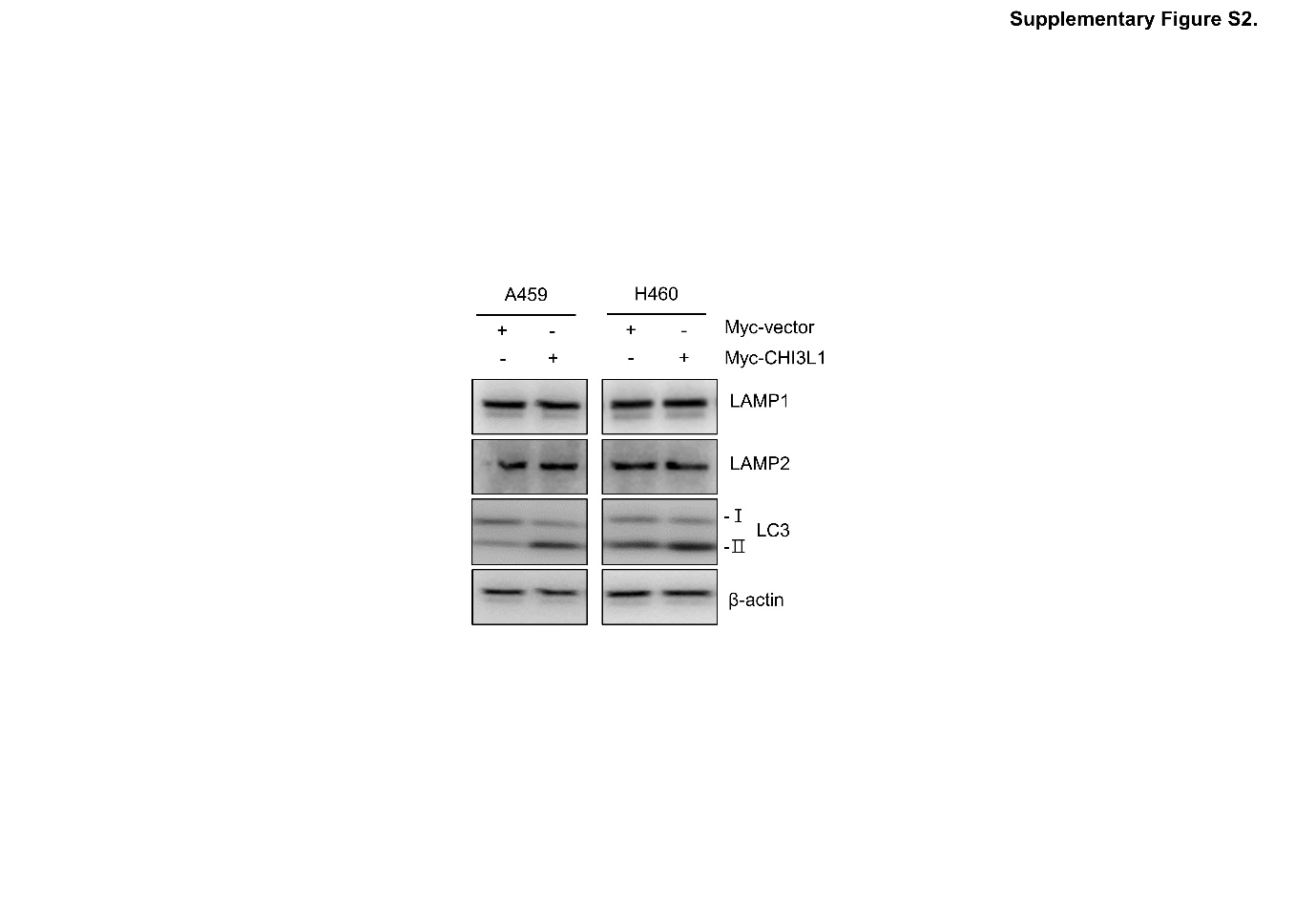
**

**Supplementary Figure S2. CHI3L1 does not affect lysosome-related protein expression.** A549 and H460 cells were transfected with either Myc-vector or Myc-CHI3L1 for 24 h. The expression of lysosome-related proteins such as LAMP-1 and LAMP-2 and LC3 protein levels were evaluated by Western blotting.


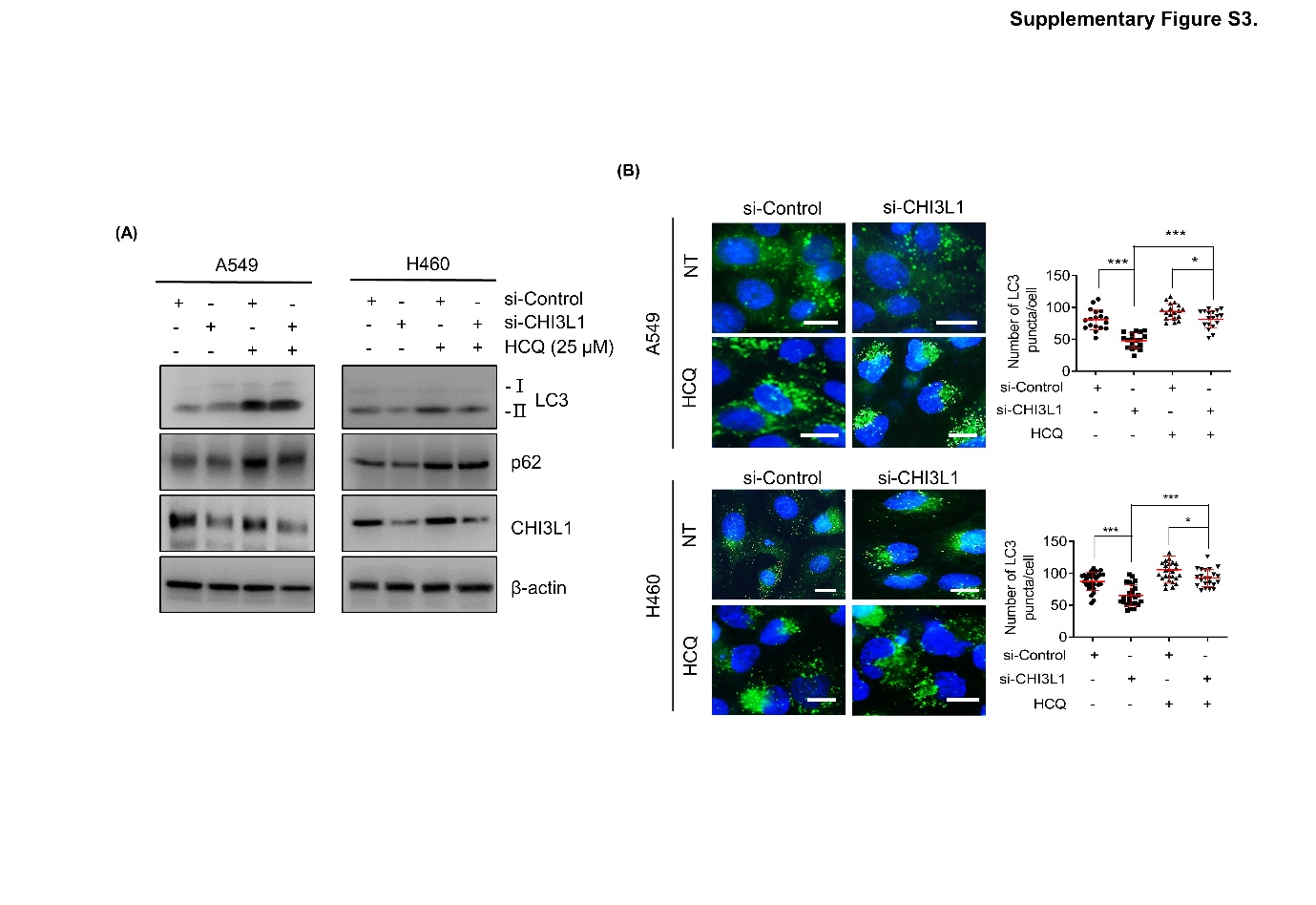


**Supplementary Figure S3. Depletion of CHI3L1 attenuates autophagic flux in human lung cancer cells.** (**A**) A549 and H460 cells were transfected with either siRNA Control or CHI3L1 siRNA for 48 h with or without HCQ (25 μM) for 6 h. The expression of LC3 and p62 levels were evaluated by Western blotting. (**B**) The cells were fixed, permeabilized and then stained with LC3 antibody. Using fluorescent microscopy, LC3 puncta formation was detected. The number of LC3 puncta per cell was calculated. The data was the average of three independent experiment and error bars were mean ± SD. ***, p<0.001; *, p<0.05. Scale bar, 10 μm.

**
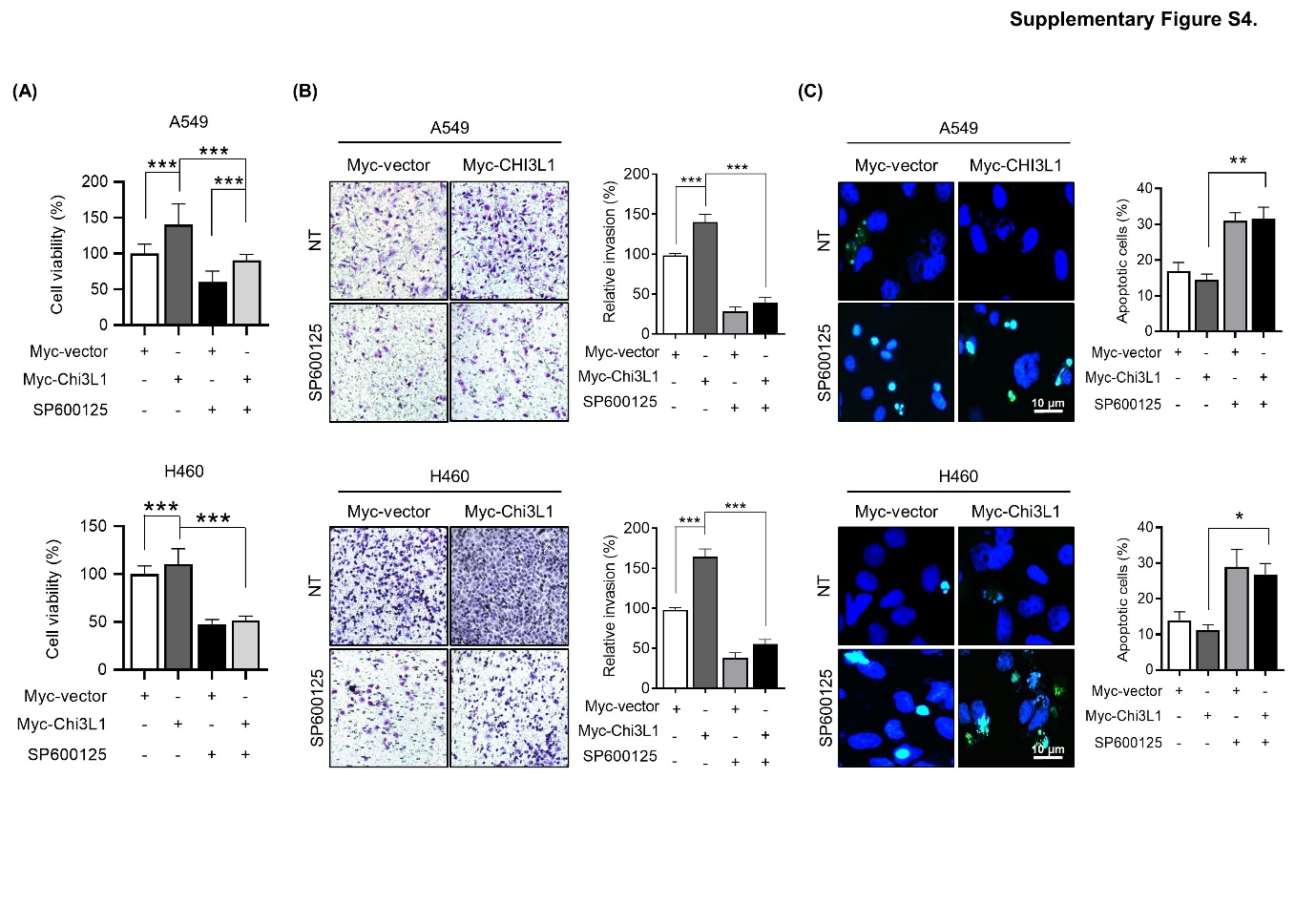
**

**Supplementary Figure S4. CHI3L1-induced autophagy via JNK activation promotes proliferation and invasion but inhibits apoptosis in lung cancer cells. .** (**A**) A549 and H460 cells were transfected with either Myc-vector or Myc-CHI3L1 for 24 h with or without JNK inhibitor, SP600125, pre-treatment (20 μM) for 2 h. The cell viability were evaluated by MTT assay. The data were expressed as the mean ± S.D. of three experiments. ***, *p* < 0.001 (**B**) The transfected cells were plated into the gelatin-coated upper chamber of a 24-well format trans-well. After 24 h of incubation, the migrated cells were stained and photographed under a microscope. The data were expressed as the mean ± S.D. of three experiments. ***, *p* < 0.001 (**C**) The transfected cells were fixed and permeabilized. Using fluorescent microscopy, TUNEL positive cells were detected. The number of apoptotic cells were calculated. The data was the average of three independent experiment and error bars were mean ± SD. **, p<0.01; *, p<0.05. Scale bar, 10 μm.

**
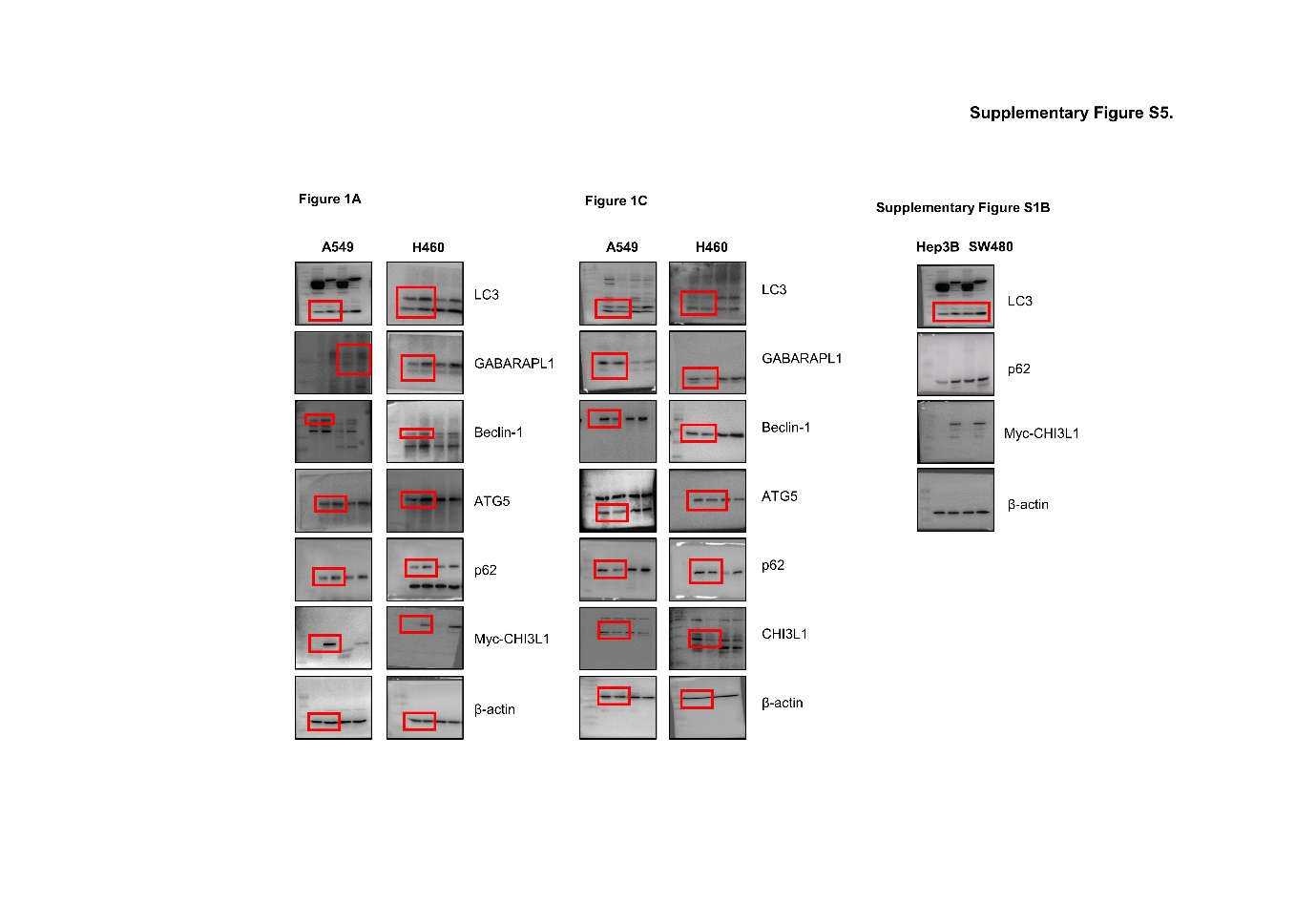

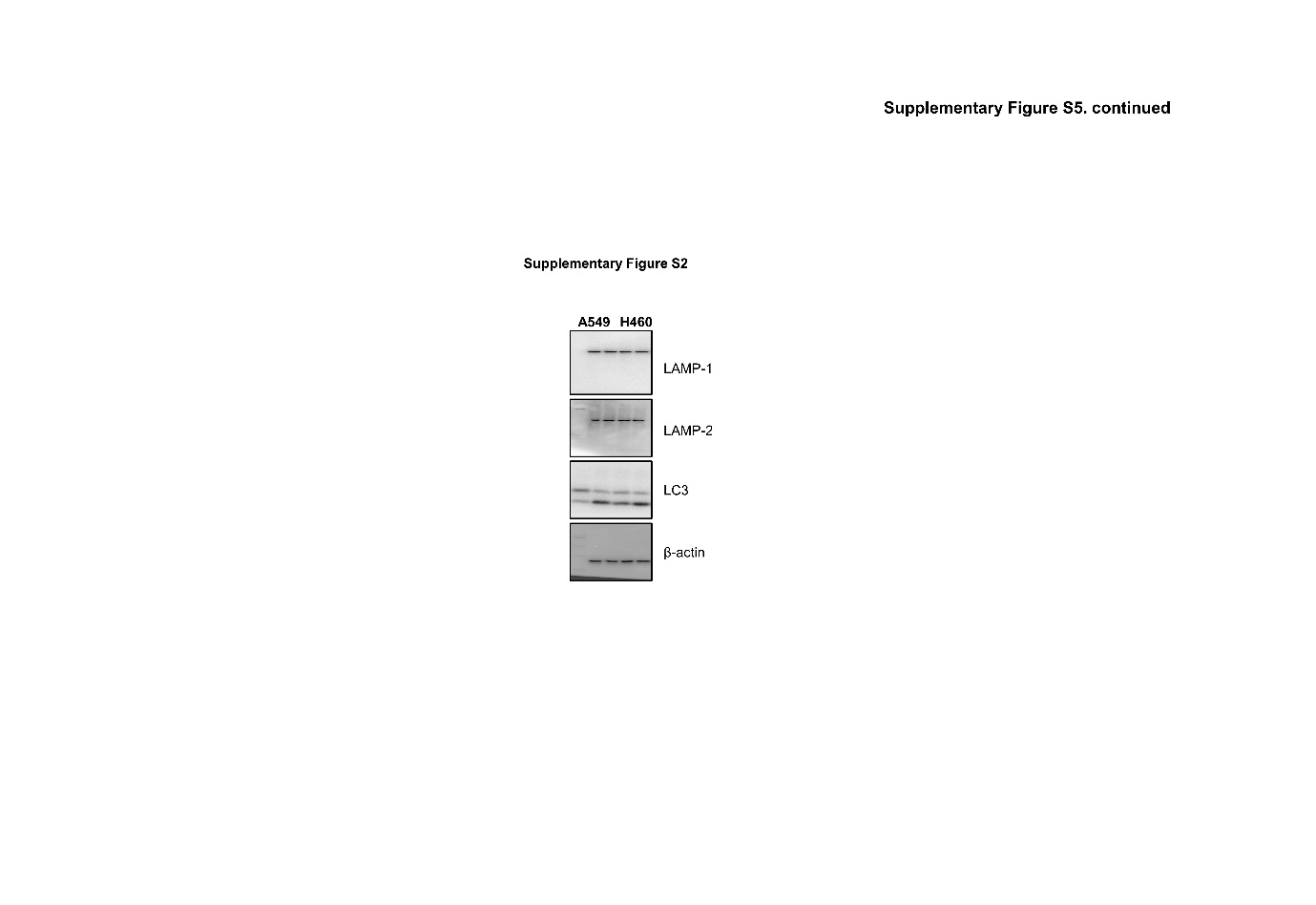

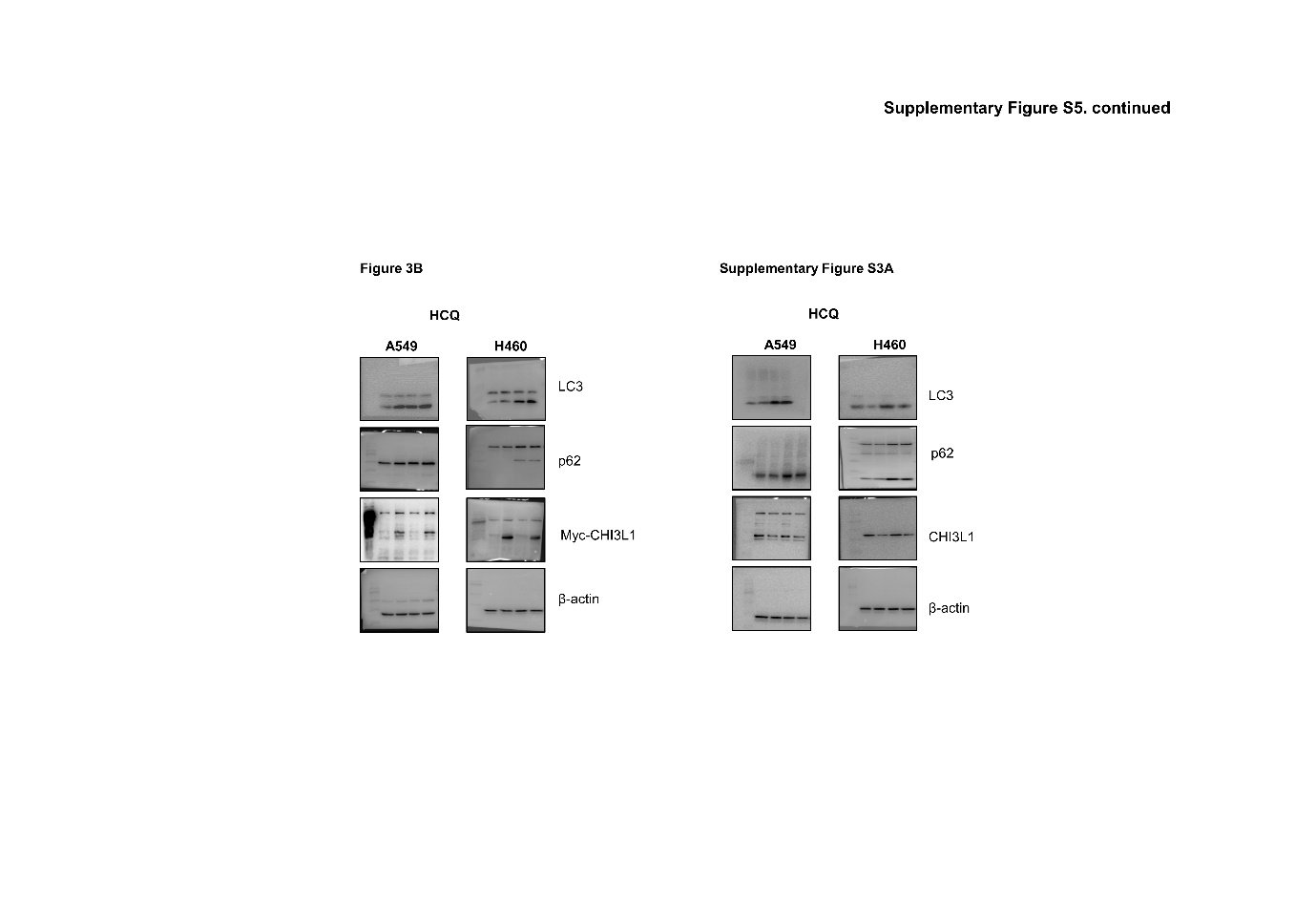

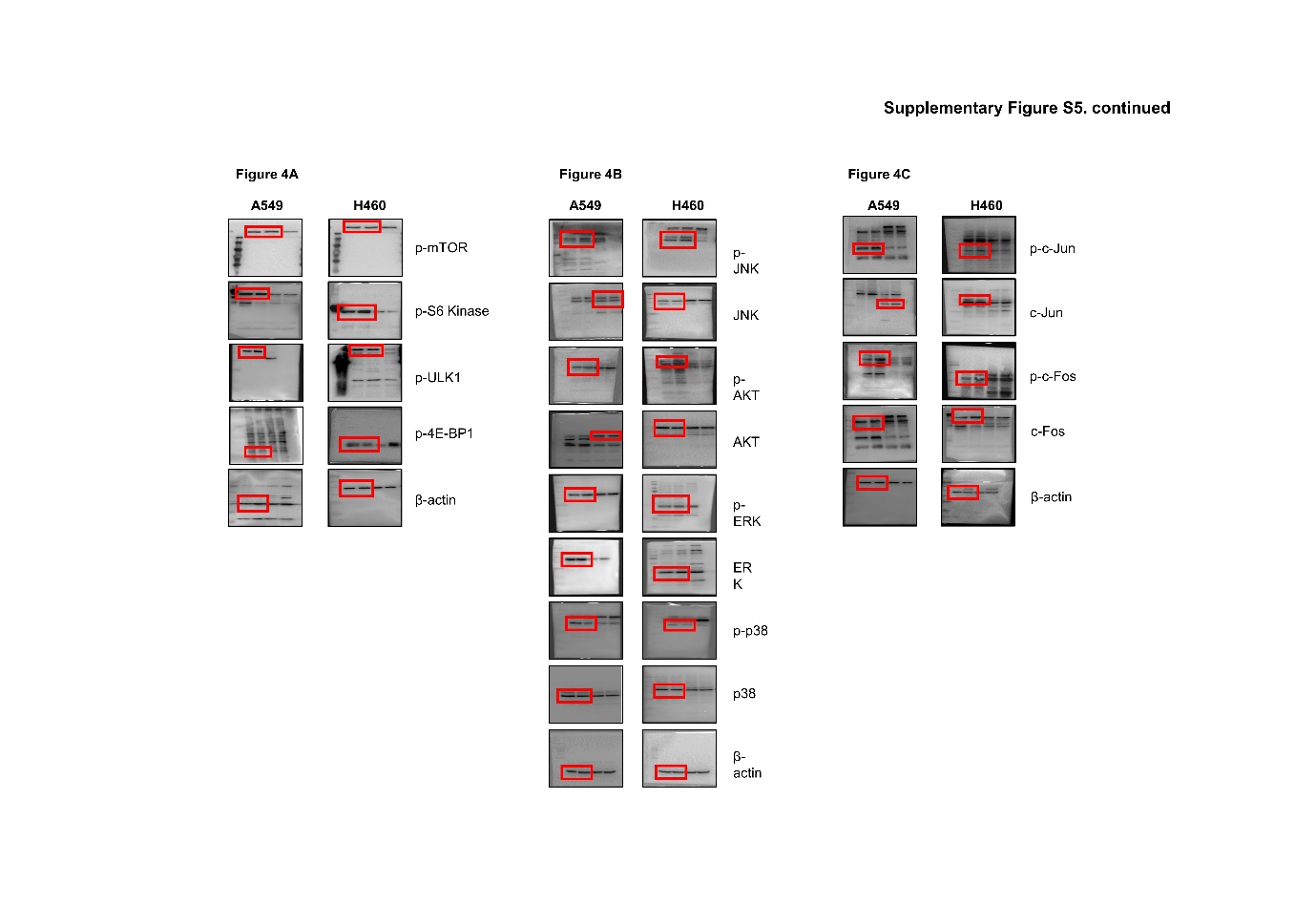

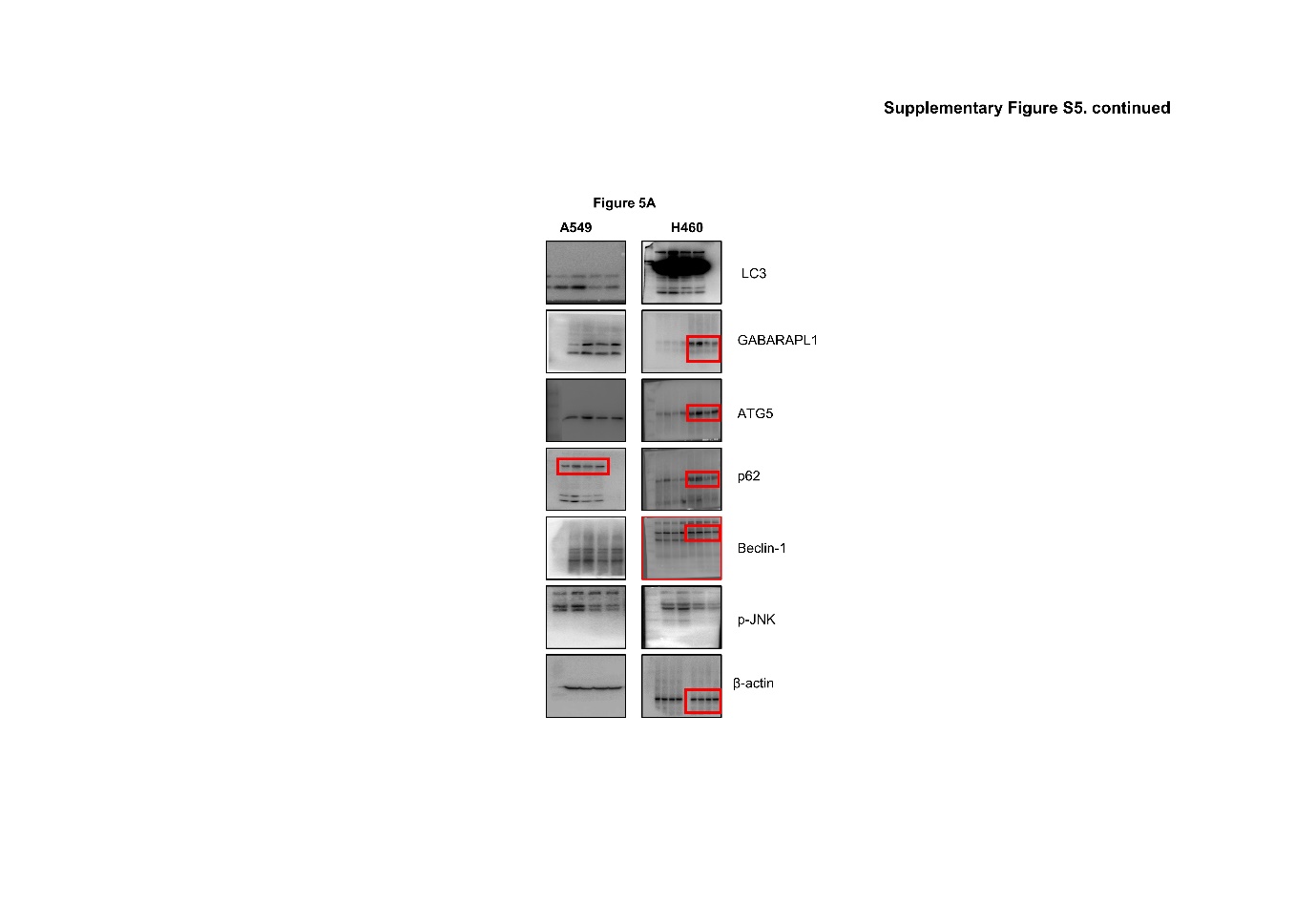

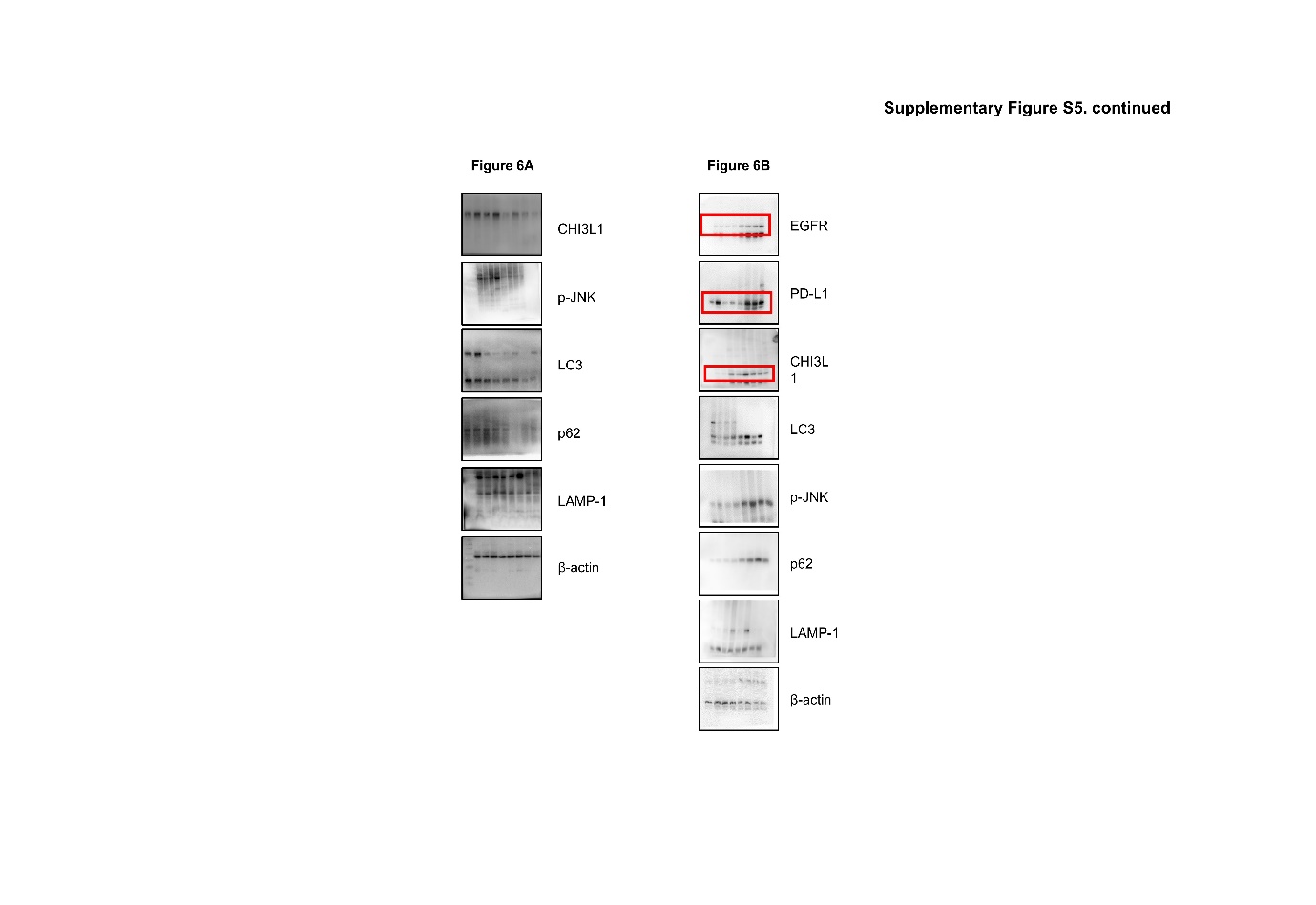
**

**Supplemental figure S5. The whole Western blot.**
